# Construction of the miRNA/Pyroptosis-Related Molecular Regulatory Axis in Abdominal Aortic Aneurysm: Evidence From Transcriptome Data Combined With Multiple Machine Learning Approaches Followed by Experiment Validation

**DOI:** 10.1155/2024/1429510

**Published:** 2024-10-30

**Authors:** Yongchao Su, Chuangang Lu, Shuchen Chen

**Affiliations:** ^1^Department of Thoracic Surgery, Fujian Medical University Union Hospital, Fuzhou 350001, Fujian Province, China; ^2^Department of Cardiothoracic Surgery, Sanya Central Hospital (The Third People's Hospital of Hainan Province), Sanya 572000, Hainan Province, China

**Keywords:** abdominal aortic aneurysm, cytosolic DNA sensing pathway, hsa-miR-331-3p, machine learning algorithms, pyroptosis, TNF

## Abstract

**Background:** Abdominal aortic aneurysm (AAA) represents a permanent and localized widening of the abdominal aorta, posing a potentially lethal risk of aortic rupture. Several recent studies have highlighted the role of pyroptosis, a pro-inflammatory programed cell death, as critical molecular regulators in AAA occurrence, progression, and rupture. However, the potential effects of pyroptosis in AAA and its upstream microRNA (miRNA) have not been comprehensively clarified.

**Methods:** Through a search of the gene expression omnibus (GEO) database, the expression profiles of mRNAs (GSE7084, GSE57691, and GSE98278) and miRNAs (GSE62179) and corresponding clinical features were downloaded, respectively. Expression profiles of 15 AAA and 10 normal vascular samples were consecutively collected for in vitro experimentation and subsequent analysis. Various machine learning techniques were employed to identify hub pyroptosis-related genes (PRGs), leading to the development of a predictive model termed the PRG classifier. Quantitative real-time-polymerase chain reaction (qRT-PCR), western blot (WB), and enzyme-linked immunosorbent assay (ELISA) were used to confirm the expression of the hub PRGs. The diagnostic and predictive capabilities of the model were comprehensively evaluated in GEO and hospital cohorts. Then, the crucial immune cell infiltration and molecular pathways implicated in the initiation and rupture of AAA and their association with pyroptosis were explored. Lastly, a miRNA/hub pyroptosis-related molecular regulatory axis was constructed using the TargetScan dataset, which was further explored through loss-of-function assays.

**Results:** Differential analysis, enrichment score analysis, and principal component analysis (PCA) revealed that pyroptosis-related molecules were significantly involved in the occurrence of AAA. Utilizing multiple machine learning algorithms, eight key PRGs (cysteinyl aspartate specific proteinase [CASP]1, infiltrating lymphocyte [IL]1B, IL18, IL6, NOD-, LRR- and pyrin domain-containing protein [NLRP]1, NLRP2, NLRP3, and tumor necrosis factor [TNF]) were integrated to establish a PRG classifier. Demonstrating robust diagnostic capabilities (area under curve [AUC] > 0.90), the PRG classifier provided clinical insights across two GEO datasets and effectively differentiated small AAA from large AAA, elective stable AAA (eAAA), and ruptured AAA (rAAA), respectively. qRT-PCR, WB, and ELISA verified the mRNA and protein expression of the hub PRGs. Notably, in hospital cohorts, a substantial positive link was unveiled between the PRG classifier and AAA risk factors (hypertension history, diastolic pressure, triglyceride levels, and aneurysm diameter). Furthermore, immune cell infiltration and functional enrichment analysis revealed significant associations of the PRG classifier/PRGs with M2 macrophage infiltration, activated dendritic cells, and enrichment scores of the cytosolic deoxyribonucleic acid (DNA) sensing pathway and tryptophan metabolism, potentially mediating AAA onset and rupture. Finally, based on 90 differentially expressed miRNAs (DEmiRNAs) and eight hub PRGs through TargetScan dataset, a hsa-miR-331-3p/TNF regulatory axis was constructed, wherein upregulation of hsa-miR-331-3p expression significantly reduced TNF and CASP1 protein levels.

**Conclusion:** A predictive model (PRG classifier) incorporating eight PRGs through multiple machine learning algorithms was developed and validated. This model may stand as a potent tool for diagnosing AAA and assessing disease severity. The identification of the cytosolic DNA sensing pathway and the hsa-miR-331-3p/TNF interaction axis may represent crucial targets for AAA treatment, offering deeper insights into its potential pathogenesis.

## 1. Introduction

Abdominal aortic aneurysm (AAA) presents as a prevalent vascular ailment marked by localized dilation and weakening of the abdominal aorta, reaching a diameter of 3 cm or greater, surpassing the normal diameter by over 50% [1]. AAA naturally progresses, posing a significant risk of irreversible aneurysmal growth and unpredictable rupture, often resulting in mortality rates reaching 80% [2]. Global reports indicate a 12% increase in AAA-related mortality over the last 20 years, presently claiming around 200,000 lives annually [3]. Typically asymptomatic until enlargement occurs, AAA remains difficult to treat promptly before rupture or mortality [4]. Early diagnosis of AAA prior to rupture, along with continuous monitoring of its biological activity, holds the potential to mitigate associated mortality risks. Currently, open surgical repair and endovascular stent graft therapies are the primary treatment modalities available. Due to the surgical risks involved, clinical practice typically reserves open surgery for patients with AAAs exceeding 5.5 cm and at risk of rupture [5]. Research indicates that by slowing down the increase in aortic diameter by 50%, the annual rates of aortic reconstruction surgery for AAA could potentially be halved [6]. However, an approved and effective pharmacotherapy for limiting and preventing AAA progression and rupture is lacking. Thus, there is a pressing need to identify novel biomarkers in the diagnosis of AAA, determination of individualized risk, discovery of therapeutic targets, and improvement of the understanding of pathogenesis.

Pathophysiologically, progressive depletion, and dysfunction of vascular smooth muscle cells (VSMCs), essential components of the vascular wall responsible for maintaining vessel function, are central to AAA formation and development [7, 8]. VSMC loss, attributed to various forms of cell demise, such as apoptosis, autophagy, and necrosis, has been involved in mediating AAA formation [9]. In recent years, pyroptosis, a programed cell death specifically dependent on Caspase activity, accompanied by the release of multiple inflammatory cytokines, has also been found to contribute to AAA [10]. Observational studies found that compared with non-AAA patients, the mRNA expression levels of NOD-, LRR- and pyrin domain-containing protein (NLRP)3, Caspase-1, and infiltrating lymphocyte (IL)-1*β* in the circulating white blood cells of AAA patients were increased [11]. Gao et al. [12] demonstrated that gastrin D (GSDMD), an essential protein in pyroptosis, exhibits upregulation in VSMCs within aortic aneurysms and vascular dissections. This upregulation promotes the synthesis of putrescine, exacerbating AAA progression. These findings indicate a significant involvement of pyroptosis in AAA occurrence and progression. Nonetheless, the potential effects of many pyroptosis molecules in AAA remain largely unclear. Therefore, a thorough comprehension of the potential functions of pyroptosis molecules is profoundly significant for advancing early AAA diagnosis, personalized management, and the development of potentially applicable drugs for AAA patients.

Prior genomic investigations have revealed that more than 98% of the human genome is actively transcribed as noncoding ribonucleic acids (ncRNAs) [13]. Traditionally, this ncRNA family is broadly categorized into two groups according to molecular size: small ncRNA (e.g., microRNA [miRNA]; length <200 nt) and long noncoding RNA (lncRNA; length >200 nt) [14]. Unlike protein-coding RNAs, miRNAs exhibit more specific expression patterns, offering a vast yet largely unexplored reservoir of potential molecular drivers of human diseases. They represent a promising new class of biomarkers for conditions like AAA. Accumulating evidence has revealed that miRNA can regulate pyroptosis-related molecules in various ways and contribute to the onset and progression of cardiovascular diseases. Using oxidized low-density lipoprotein-stimulated human THP-1 derived macrophage, Wang et al. [15] found that miRNA-9 suppresses NLRP3 inflammasome activation and mitigates pyroptosis via the JAK1/STAT signaling pathway, thus attenuating atherosclerosis-related inflammation. Li et al. [16] experimentally demonstrated that miRNA-30c-5p can alleviate the pyroptosis of human aorta epithelial cells by inhibiting FOXO3 expression. However, the specific miRNAs implicated in the regulation of AAA pyroptosis remain ambiguous. Hence, it is crucial to investigate the upstream miRNAs that regulate pyroptosis molecular pathways in AAA to advance the development of novel pharmacological therapies.

The availability of high-throughput RNA-sequencing (RNA-seq) datasets provides an unprecedented opportunity to apply advanced machine learning algorithms to discover novel diagnostic, predictive, and therapeutic biomarkers. In the present study, available transcriptomic profiling in gene expression omnibus (GEO) datasets was systematically reviewed to identify hub pyroptosis-related genes (PRGs) and construct a PRG classifier using multiple machine learning algorithms. Fifteen AAA and 10 normal vascular samples were consecutively collected for in vitro experiments. Then, the diagnostic and predictive capacities of the model were thoroughly assessed in GEO and hospital cohorts. The pivotal immune cells infiltrating and underlying pathways implicated in the onset and rupture of AAA, as well as their relationship with pyroptosis, were investigated. Finally, a miRNA/hub pyroptosis-related molecular regulatory axis was constructed based on the TargetScan dataset, and this regulatory axis was further investigated through loss-of-function assays.

## 2. Materials and Methods

### 2.1. Dataset Collection, Sample Selection, and Preprocessing

A systematic search was carried out in the GEO (http://www.ncbi.nlm.nih.gov/geo) database to discern relevant microarray data of AAA. During the retrieving process, the species type *Homo sapiens* was set as a filter, resulting in the inclusion of four datasets (GSE7084, GSE57691, GSE98278, and GSE62179) for both qualitative and quantitative analysis. As for the three expression profiling datasets, 19 samples (10 normal and 9 AAA samples) were obtained in GSE7084, 59 samples in GSE57691 (normal [*n* = 10], small AAA [*n* = 20, mean maximum aortic diameter = 54.3 ± 2.3 mm], and large AAA samples [*n* = 29, mean maximum aortic diameter = 68.4 ± 14.3 mm]), and 48 samples in GSE98278 (elective stable AAA [eAAA] [*n* = 31] vs. ruptured AAA [rAAA] [*n* = 17] AAA and intermediate size AAA [*n* = 15, mean maximum aortic diameter ≤55 mm] vs. large AAA [*n* = 16, mean maximum aortic diameter >70 mm]). In terms of the miRNA profiling dataset, six samples (two normal and four AAA SMC) were retained in GSE62179. Robust multiarray average analysis was used for microarray data, encompassing background correction, quantile normalization, and summarization [17]. Furthermore, 33 PRGs were compiled from published studies [18] (Supporting Information 1).

### 2.2. Landscape of Expression Variation in PRGs in AAA

Differential analysis was executed on the expression of PRGs in the GSE7084 and GSE57691 datasets, comparing control and AAA groups, respectively. The results were visualized using box plots. The single-sample gene set enrichment analysis (ssGSEA) algorithm [19] was employed to calculate enrichment scores of pyroptosis, comparing the scores between the control and AAA groups. Finally, principal component analysis (PCA) [20], utilizing PRGs expression levels, was executed on the GSE7084 and GSE57691 datasets.

### 2.3. Discovery of Hub PRGs and Development of a PRG Classifier

To identify robust hub PRGs, machine learning techniques with fivefold cross-validation based on disease status were applied to the GSE7084 and GSE57691 datasets. This included modified least absolute shrinkage and selection operator (LASSO) penalized regression, random forest (RF), and support vector machine (SVM) to choose feature variables. The hub PRGs were derived from the overlapping of the results obtained from SVM, RF, and LASSO.

Artificial neural network (ANN) models were constructed and trained, comprising input, hidden, and output layers. Within each hidden node, the ReLU (rectified linear unit) activation function was employed. The output layer consisted of two nodes (O1 and O2, representing normal and AAA, respectively). A softmax function was applied to each node, designating y2 (probability of AAA; i.e., the O2 node) as Y. Cross-entropy error was utilized as the loss function (E), and the weight values were optimized using the Adam method (learning rate, 0.001; epochs, 1000) [21]. Following training, the weights of the nodes (“NeuralNetworkWeight”) were utilized to compute the PRG score. The formula for calculating the PRG score based on hub PRGs is as follows:

  $$\begin{array}{c} \text{Neural AAA }=\Sigma \left(\text{Gene expression }\times \text{neural network weight}\right). \end{array}$$

### 2.4. Diagnostic Value, Risk Stratification, and Clinical Usefulness of PRG Classifier

Receiver operating characteristic (ROC) analysis was executed to examine the diagnostic capability of the PRG classifier with AAA as the endpoint in the GSE7084 and GSE57691 datasets. Furthermore, the PRG score was compared between small AAA and large AAA in the GSE57691 cohort, between eAAA and rAAA in the GSE98278 cohort, and between intermediate AAA and large AAA in the GSE98278 cohort. ROC analyses were also conducted to evaluate the ability of the PRG classifier to distinguish small AAA from large AAA, eAAA from rAAA, and intermediate AAA from large AAA, respectively. Ultimately, decision curve analysis (DCA) was employed to determine the clinical practicality of the PRG classifier.

### 2.5. Design, Study Setting, and Population

A prospective cohort study was conducted among 15 patients with AAA after open surgery at the Department of Vascular Surgery from March 9, 2022, to December 24, 2023. Criteria for inclusion in this study are as follows: (1) participants aged over 18 years and; (2) diagnosis of AAA confirmed via computer tomography angiography. Ethical approval was acquired from the Ethical Guideline of the Committee on Human Experimentation of The Third People's Hospital of Hainan Province (reference: 2023-10-22). Prior to sample and data collection, written informed consent was acquired from the relatives of eligible patients.

### 2.6. Tissue Samples and Quantitative Real-Time-Polymerase Chain Reaction (qRT-PCR)

Human AAA and normal samples were acquired from surgical patients and preserved at −80°C for subsequent in vitro experiments. Total RNA was isolated from AAA and normal samples, as well as MOVAS cells, as per the provided guidelines using Trizol (Invitrogen). Reverse transcription of RNA was executed utilizing the RevertAid RT Reverse Transcription Kit (Thermo Scientific). Quantitative PCR was conducted utilizing PowerUp SYBR Green Master Mix (Thermo Scientific), with GAPDH serving as the internal standard. Quantitative reverse transcription-PCR was executed utilizing the ABI 7500 real-time PCR system (Applied Biosystems, Foster City, CA, United States). Fold change in gene expression was examined using the 2^−*ΔΔ*Ct^ method. Gene-specific PCR primers are provided in Supporting Information 2.

Following the expression levels of hub PRGs determined through qRT-PCR testing, the PRG score was calculated. Using the median score of PRGs as a threshold, each case was then classified into either the low or high PRG groups.

### 2.7. Data Collection and Correlation of PRG Classifier With Clinical Characteristics

Clinical data were collected from electronic medical records. These data encompass age, height, sex, body mass index (BMI), weight, hypertension history, diabetes history, smoking history, fasting blood glucose (FBG), systolic pressure at admission, diastolic pressure at admission, triglyceride at admission, cholesterol at admission, and aneurysm diameter. The link between the PRG classifier and clinical features in hospital cohorts was investigated.

### 2.8. Analyses of Immune Cell Infiltration in AAA and Its Relationship With Hub PRGs/PRG Classifier

The CIBERSORTx tool and ssGSEA algorithm [22] were utilized to infer the relative abundance of infiltrating immune cells. The differential composition of immune cells between the normal and AAA groups, as well as between the eAAA and rAAA subgroups, was examined. Spearman's correlation analyses were executed to explore the link between hub PRGs/PRG classifier and immune cells.

### 2.9. Discovery of Potential Mechanisms in the Development and Progression of AAA and Its Relationship With Hub PRGs/PRG Classifier

ssGSEA analysis was performed on several representative gene sets (immune-related pathways) [23]. Then, the gene set variation analysis (GSVA) [24] was carried out to discern the signaling pathways according to the Kyoto Encyclopedia of Genes and Genome (KEGG) gene sets (c2.cp.kegg.v7.4.symbols.gmt) obtained from the Molecular Signatures Database. In the analysis of signaling pathways, the differential enrichment score between the normal and AAA groups, as well as between the eAAA and rAAA subgroups, was investigated. Following this, correlation analysis was executed to further elucidate the link between the PRG classifier/hub PRGs and some key biological pathways.

### 2.10. Construction of the miRNA/Pyroptosis-Related Molecular Regulatory Axis

Differentially expressed miRNAs (DEmiRNAs) (GSE62179 dataset) between individuals with AAA and healthy control samples were identified. Pairs of DEmiRNAs and hub PRGs were predicted utilizing target miRNA data obtained from the TargetScan databases.

### 2.11. Cell Culture and Treatment

The mouse smooth muscle cell (SMC) line MOVAS was sourced from the American Type Culture Collection (ATCC, Manassas, VA, United States). Cells were grown in Petri dishes with Dulbecco's Modified Eagle's Medium (DMEM) comprising 10% fetal bovine serum. Ang II is a commonly utilized stimulant for inducing AAA formation. Cultures were treated with DMEM containing 15 µM Ang II (Sigma–Aldrich, St. Louis, MO, United States) and incubated for 24 h.

### 2.12. Cell Transfection

Transfectants, including miR-331-3p mimic and negative control (NC), were sourced from RiboBio (Guangzhou, China). MOVAS were seeded into 96-well plates and underwent transfection with miR-331-3p mimic and NC vectors utilizing Lipofectamine 2000 (Invitrogen) following the provided guidelines.

### 2.13. Enzyme-Linked Immunosorbent Assay (ELISA)

Total protein extraction was executed utilizing the Total Protein Extraction Kit (Beyotime). Following centrifugation at 3000× *g* for 8 min within 40 min, the expression levels of inflammatory cytokines IL1B, IL18, IL6, and tumor necrosis factor (TNF) were measured in the homogeneous supernatants of samples. This was achieved using the corresponding ELISA kits following the provided guidelines.

### 2.14. Western Blotting (WB) Analysis

Total proteins were isolated and the protein concentration was examined utilizing the BCA Protein Assay Kit (Thermo Fisher Scientific). Subsequent to this, protein samples (30 μg) underwent 10% SDS-PAGE gel electrophoresis, followed by transfer onto nitrocellulose membranes (Invitrogen). These membranes underwent incubation with anti-cysteinyl aspartate specific proteinase (CASP)1 (1 : 1000; Abcam), anti-NLRP1 (1 : 1000; Abcam), anti-NLRP2 (1 : 1000; Abcam), anti-NLRP3 (1 : 1000; Abcam), anti-TNF (1 : 1000; Abcam), and anti-GAPDH (1 : 5000; Proteintech, Wuhan, China) at 4°C for 12 h. The membranes were exposed to HRP-IgG antibody (1:5000; Proteintech).

### 2.15. Statistical Analysis

Statistical analysis was executed utilizing GraphPad Prism 9, SPSS 22, and R software (R v4.3.1). Continuous variables were compared utilizing Student's *t*-tests, Mann–Whitney, Wilcoxon, or Kruskal–Wallis tests with two-tailed analyses based on the distribution of the variables. Categorical variables were tested utilizing the *χ*^2^ or Fisher's exact tests. The threshold for statistical significance was established at *p* < 0.05 unless specific *p*-values were provided.

## 3. Results

### 3.1. Landscape of Expression Variation in PRGs in AAA

**Figure 1:**
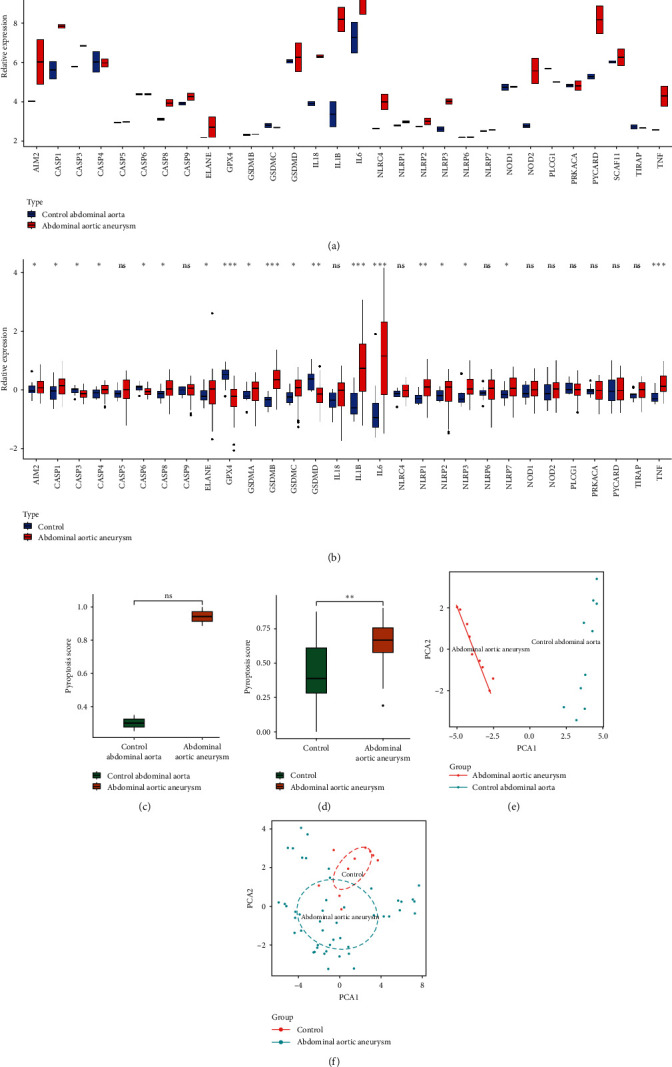
Landscape of expression variation in PRGs in AAA. (A and B) Differential expression of PRGs between AAA samples and healthy control patient samples in two GEO datasets. (A) GSE7084 datasets; (B) GSE57691 datasets. The asterisks indicate a significant statistical *p* value calculated using the Wilcoxon test (*⁣*^*∗*^*p*  < 0.05; *⁣*^*∗∗*^*p*  < 0.01; *⁣*^*∗∗∗*^*p*  < 0.001). (C and D) Comparison of PRGs score between AAA samples and healthy control patient samples in two GEO datasets. (C) GSE7084 datasets; (D) GSE57691 datasets. The asterisks indicate a significant statistical *p* value calculated using the Wilcoxon test (*⁣*^*∗*^*p*  < 0.05; *⁣*^*∗∗*^*p*  < 0.01; *⁣*^*∗∗∗*^*p*  < 0.001). (E and F) Principal component analysis for the expression profiles of PRGs to distinguish AAA patients from healthy control patients in two AAA cohorts. (E) GSE7084 datasets; (F) GSE57691 datasets. AAA, abdominal aortic aneurysm; GEO, gene expression omnibus; IL, infiltrating lymphocyte; PCA, principal component analysis; PRGs, pyroptosis-related genes; TNF, tumor necrosis factor.

The mRNA expression levels of PRGs between control and AAA groups were examined, revealing that most PRGs exhibited differential expression in the GSE7084 (Figure 1A) and GSE57691 datasets (Figure 1B). ssGSEA results demonstrated that the enrichment scores of pyroptosis were notably increased in the AAA group than in the control group (Figure 1C,D). PCA demonstrated that the expression of these PRGs could effectively differentiate between AAA patients and control patients in both the GSE7084 (Figure 1E) and GSE57691 datasets (Figure 1F). These analyses underscored the pivotal role of PRG expression imbalance in the occurrence of AAA.

### 3.2. Identification and Validation of Hub PRGs and Development of a PRG Classifier

**Figure 2:**
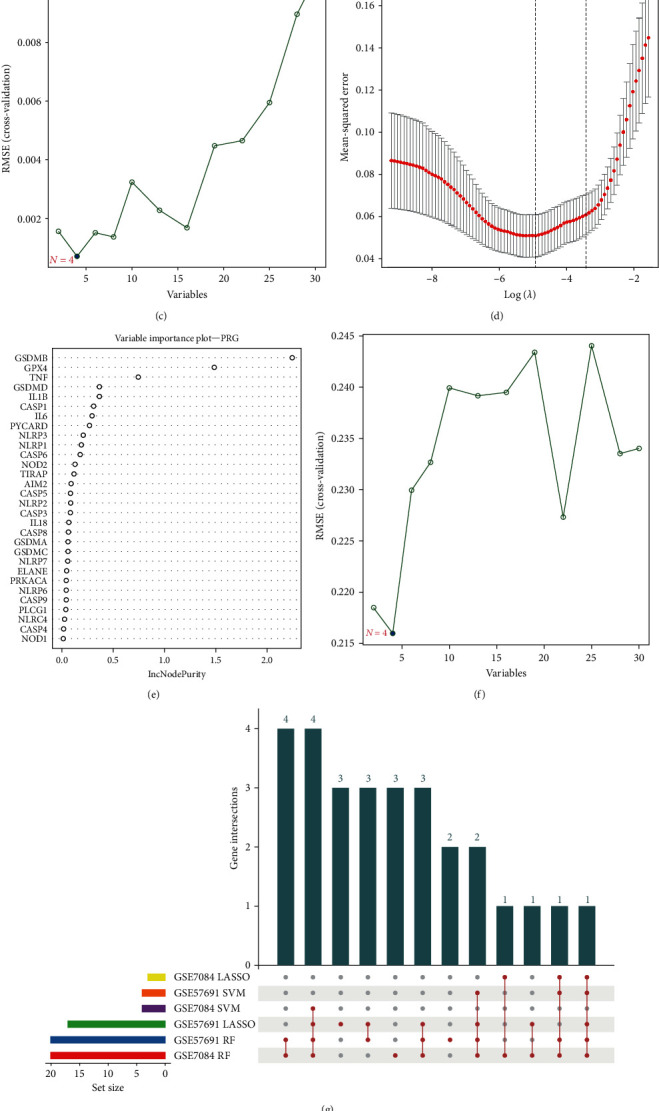
Identification of hub PRGs and construction of a PRG classifier based on machine learning algorithms (including LASSO, RF, and SVM, ANN) with fivefold cross-validation. (A and D) Modified LASSO was used to identify candidate PRGs in two AAA datasets. The *Y*-axis shows mean square error and the *X*-axis is log (*λ*). Dotted vertical lines represent minimum and 1 standard error values of *λ*. The genes selected at minimum standard error values of *λ* were finally used for further analysis. (A) GSE7084 datasets (*N* = 3); (D) GSE57691 datasets (*N* = 17). (B and E) RF was used to select candidate PRGs with in two AAA datasets and ranked important variables by IncNodePurity. (B) GSE7084 datasets; (E) GSE57691 datasets. SVM algorithm was applied to screen candidate PRGs. (C and F) SVM algorithm was applied to screen candidate PRGs. The blue dots indicated the lowest error rate and the highest precision when genes are this number in GSE57691 (C) and GSE57691 (F) datasets. (G) An UpSet diagram exhibited the interaction result of LASSO, RF, and SVM in two AAA datasets. (H) The visualization of ANN. The neural network contains six input layers, three hidden layers, and two output layers. AAA, abdominal aortic aneurysm; ANN, artificial neural network; LASSO, least absolute shrinkage and selection operator; PRGs, pyroptosis-related genes; RF, random forest; SVM, support vector machine.

To identify hub PRGs during AAA in the training cohort (GSE7084 and GSE57691), the expression data of 33 PRGs were input into multiple machine learning models. The LASSO was utilized to shrink and select candidate PRGs in the GSE7084 (Figure 2A) and GSE57691 datasets (Figure 2D). RF, an ensemble of tree-based classifiers, was constructed, and feature variables were selected by means of a “randomForest” package, employing a minimum error regression tree. IncNodePurity was utilized to rank the importance of variables (Figure 2B,E). SVM was then employed to identify candidate PRGs according to minimum root mean square error (RMSE; Figure 2C,F). By intersecting the results of LASSO, RF, and SVM, eight hub genes (CASP1, IL1B, IL18, IL6, NLRP1, NLRP2, NLRP3, and TNF) were identified, shared by four or more algorithms (Figure 2G). Utilizing these eight hub PRGs, a prognostic model named the PRG classifier was constructed.

Utilizing the expression transformation of eight hub PRGs, an ANN model was built to optimize the weights of each gene. The ANN model contains eight input layers, three hidden layers, and two output layers (Figure 2H). The PRG score was computed by the summation of “GeneExpression” × “NeuralNetworkWeight” for nine hub PRGs. The potential values ranged from 0 to 1, with detailed data available in Supporting Information 3.

### 3.3. Diagnostic Value, Risk Stratification, and Clinical Usefulness of PRG Classifier

**Figure 3:**
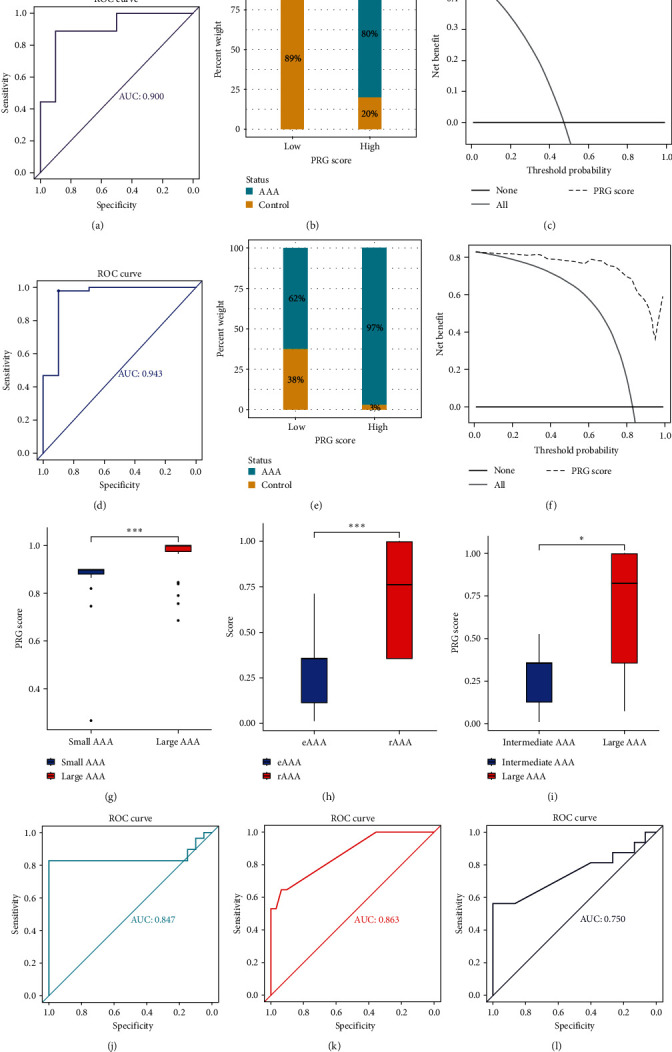
Diagnostic value, risk stratification, and clinical usefulness of PRG classifier in GEO cohorts. ROC analysis was executed to examine the diagnostic capability of the PRG classifier with AAA as the endpoint in the GSE7084 (A) and GSE57691 (D) datasets. (B and E) The distribution of AAA occurrence in PRG different subgroups in two AAA datasets. (B) GSE7084 datasets; (E) GSE57691 datasets. Decision curve analysis was applied to evaluate the clinical usefulness of PRG classifier in GSE7084 (C) and GSE57691 (F) datasets. The *Y*-axis represents the net benefit. The black line represents the hypothesis that no patients treatment. The *X*-axis represents the threshold probability. The threshold probability is where the expected benefit of treatment is equal to the expected benefit of avoiding treatment. Comparison of the PRG scores between small AAA and large AAA in the GSE57691 cohort (G), between eAAA and rAAA in the GSE98278 cohort (H), and between intermediate AAA and large AAA in the GSE98278 cohort (I). The asterisks indicate a significant statistical *p* value calculated using the Wilcoxon test (*⁣*^*∗*^*p*  < 0.05; *⁣*^*∗∗∗*^*p*  < 0.001). ROC analyses were conducted to evaluate the ability of the PRG classifier to distinguish small AAA from large AAA in GSE57691 cohort (J), eAAA from rAAA in GSE98278 cohort (K), and intermediate AAA from large AAA in GSE98278 cohort (L), respectively. AAA, abdominal aortic aneurysm; AUC, area under curve; eAAA, elective stable AAA; GEO, gene expression omnibus; PRG, pyroptosis-related gene; rAAA, rupture AAA; ROC, receiver operating characteristic.

ROC curves revealed that the PRG classifier exhibited a superior diagnostic capacity, with an area under curve (AUC) of 0.900 in the GSE7084 cohort (Figure 3A) and an AUC of 0.943 in the GSE57691 cohort (Figure 3D). As per the median PRG score, each case was sorted into low or high PRG groups. Individuals in the high-risk group highlighted a considerably elevated AAA incidence relative to the low-risk group (*p*  < 0.001 for *χ*^2^ test) in GSE7084 (Figure 3B) and GSE57691 cohorts (Figure 3E). Notably, the DCA chart showed that within the threshold probability range of 0–1.0, the net benefit rate derived from employing the PRG classifier (predictive model) exceeded that obtained by either not intervening in all patients or intervening in all patients in GSE7084 (Figure 3C) and GSE57691 cohorts (Figure 3F).

Furthermore, in the GSE57691 cohort, the PRG score exhibited a considerable increase in large AAA compared to small AAA (*p* < 0.001, Figure 3G), while in the GSE98278 cohort, it was higher in rAAA than in eAAA (*p* < 0.001, Figure 3H), and in GSE98278, it was higher in large AAA relative to intermediate AAA (*p* < 0.05, Figure 3I). The PRG classifier effectively discriminated large AAA from small AAA with an AUC of 0.847 in the GSE57691 datasets (Figure 3J), rAAA from eAAA with an AUC of 0.863 in the GSE98278 datasets (Figure 3K), and large AAA from intermediate AAA with an AUC of 0.750 in the GSE98278 datasets (Figure 3L).

### 3.4. Clinical Information From the Hospital Cohort: Validation Cohort Analysis

**Figure 4:**
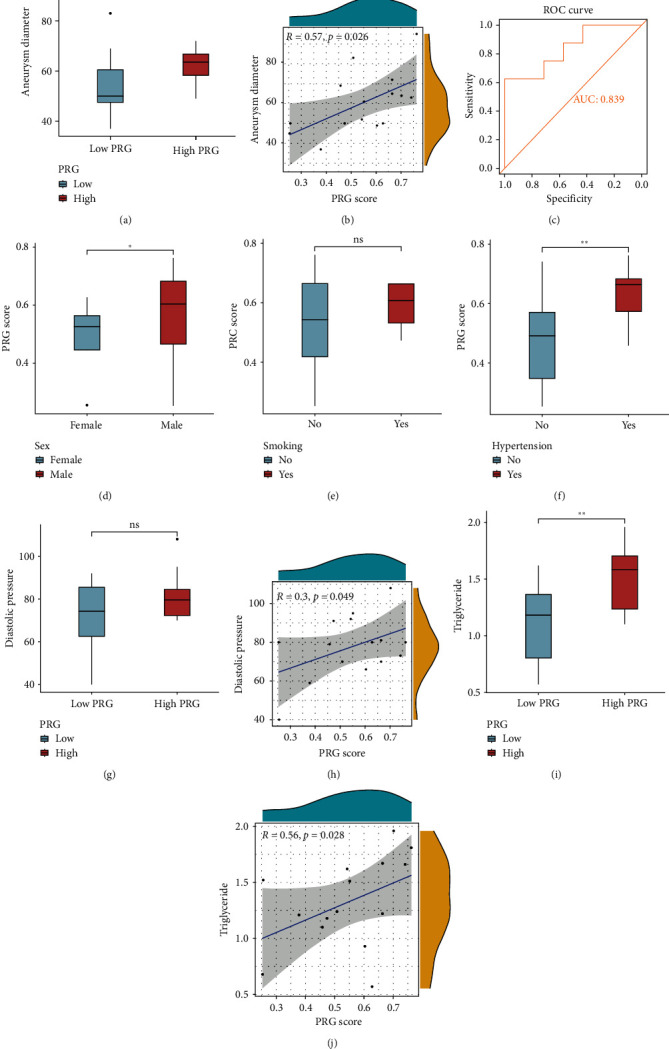
Correlation of PRG classifier with clinical characteristics in hospital cohort. (A) Comparison of the aneurysm diameter in PRG different subgroups. The asterisks indicate a significant statistical *p* value calculated using the Wilcoxon test (*⁣*^*∗*^*p*  < 0.05; *⁣*^*∗∗*^*p*  < 0.01; *⁣*^*∗∗∗*^*p*  < 0.001). (B) Correlation between PRG score and aneurysm diameter. Correlation coefficient and *p* value were calculated by Spearman's correlation analysis. (C) The ROC curve was used to evaluate the effectiveness of PRG in diagnosing aneurysm diameter. Comparison of the PRG scores based on different clinical features, including female vs. male (D), smoking history (E), hypertension history (F). Comparison of the diastolic pressure (G) and triglyceride (I) in PRG different subgroups. The asterisks indicate a significant statistical *p* value calculated using the Wilcoxon test (*⁣*^*∗*^*p*  < 0.05; *⁣*^*∗∗*^*p*  < 0.01; *⁣*^*∗∗∗*^*p*  < 0.001). Correlation between PRG score and diastolic pressure (H) and triglyceride (J). Correlation coefficient and *p* value were calculated by Spearman's correlation analysis. PRG, pyroptosis-related gene; ROC, receiver operating characteristic.

Detailed clinical data of AAA patients included in this research is summarized in Table 1. The differential analysis observed that relative to the low PRG subgroup, patients in the high PRG subgroup indicated larger aneurysm diameter (*p*  < 0.001, Figure 4A), higher triglyceride level (*p*  < 0.01, Figure 4I), higher height (*p*  < 0.05, Supporting Information 4: Figure S1), and heavier weight (*p*  < 0.05, Supporting Information 4: Figure S1). Other clinical indicators, such as diastolic pressure (Figure 4G), age (Supporting Information 4: Figure S1), BMI (Supporting Information 4: Figure S1), systolic pressure (Supporting Information 5: Figure S2), cholesterol (Supporting Information 5: Figure S2), and FBG (Supporting Information 5: Figure S2), did not yield significant statistical differences, but diastolic pressure showed a significant trend. Moreover, correlation analysis demonstrated a substantial positive association between PRG and aneurysm diameter (*R* = 0.57, *p* = 0.026; Figure 4B), diastolic pressure (*R* = 0.3, *p* = 0.049; Figure 4H), and triglyceride level (*R* = 0.56, *P* = 0.028; Figure 4J). Other clinical features were not significantly correlated with PRG (Supporting Information 4: Figure S1 and Supporting Information 5: Figure S2). Furthermore, higher PRG scores were observed in males (*p* < 0.05, Figure 4D) and hypertensive patients (*p* < 0.01, Figure 4F), while smokers (*p* > 0.05, Figure 4E) displayed elevated PRG scores that did not reach statistical significance. The ROC curve confirmed the effectiveness of PRG in diagnosing aneurysm diameter with an AUC of 0.839 (Figure 4C).

### 3.5. Analyses of Immune Cell Infiltration in AAA and Its Relationship With Hub PRGs/PRG Classifier

**Figure 5:**
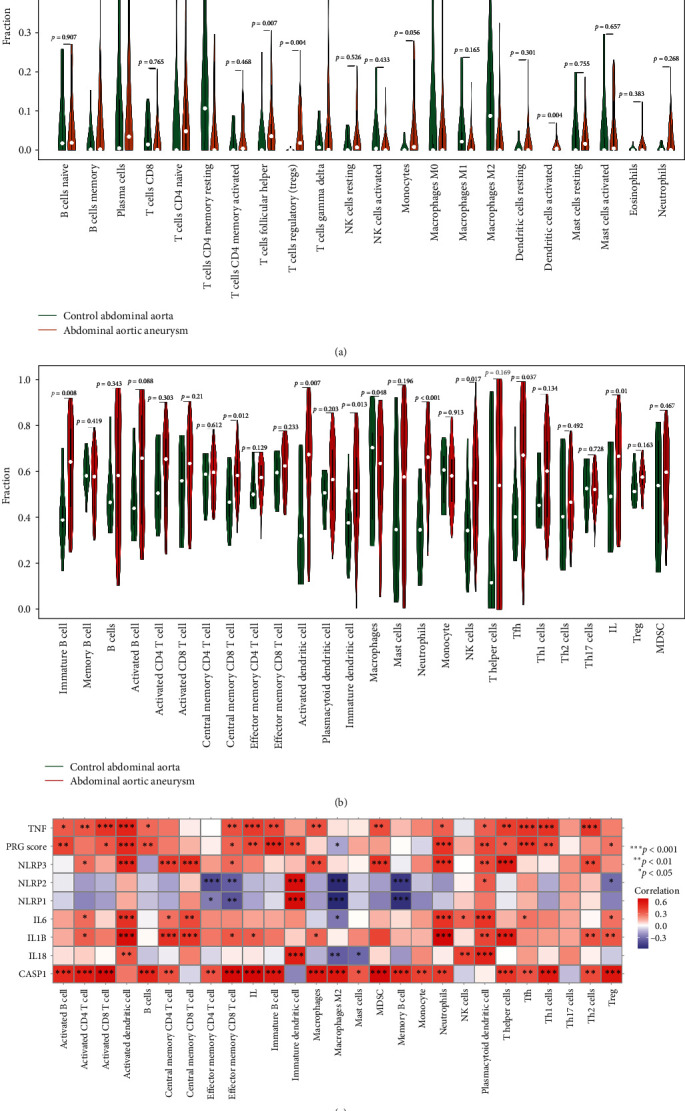
Analyses of immune cell infiltration in AAA and its relationship with hub PRGs/PRG classifier in GSE57691 cohort. Comparison of the immune cell infiltration between normal and AAA groups based on CIBERSORTx (A) and ssGSEA (B) algorithms. (C) Correlation between hub PRGs/PRG classifier and immune cells. AAA, abdominal aortic aneurysm; IL, infiltrating lymphocyte; MDSCs, myeloid-derived suppressor cells; NK, natural killer; PRG, pyroptosis-related gene; ssGSEA, single-sample gene set enrichment analysis; Tfh, T cells follicular helper; TNF, tumor necrosis factor.

**Figure 6:**
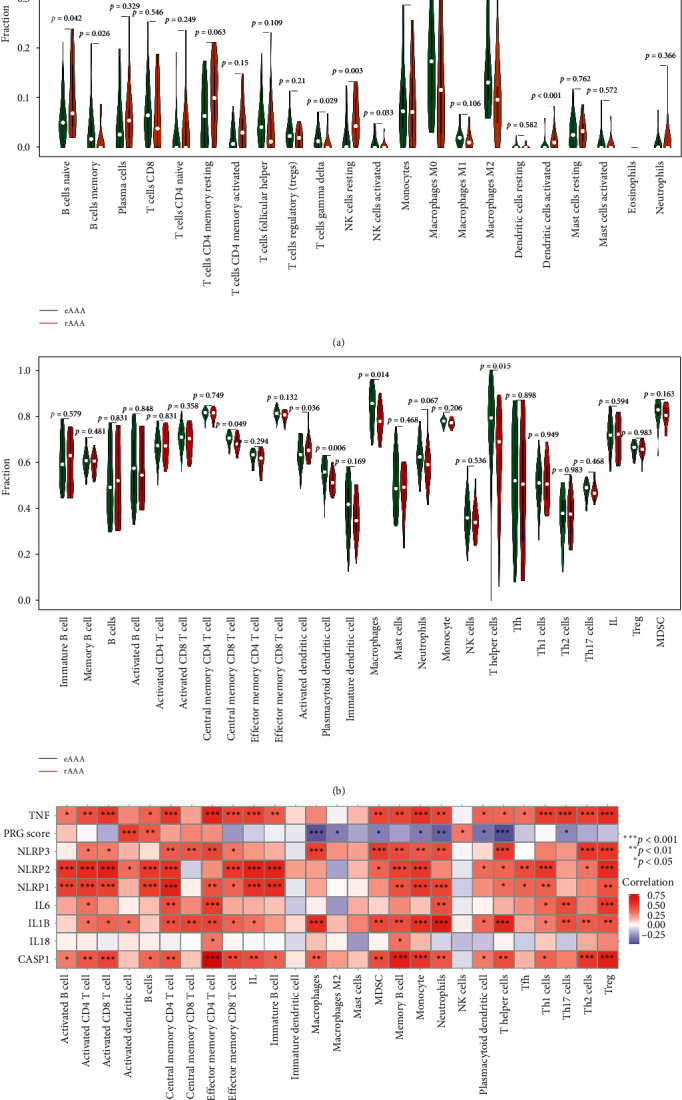
Analyses of immune cell infiltration in AAA and its relationship with hub PRGs/PRG classifier in GSE98278 cohort. Comparison of the immune cell infiltration between eAAA and rAAA subgroups based on CIBERSORTx (A) and ssGSEA (B) algorithms. (C) Correlation between hub PRGs/PRG classifier and immune cells. AAA, abdominal aortic aneurysm; eAAA, elective stable AAA; IL, infiltrating lymphocyte; NK, natural killer; PRG, pyroptosis-related gene; rAAA, rupture AAA; ssGSEA, single-sample gene set enrichment analysis; Tfh, T cells follicular helper; TNF, tumor necrosis factor.

To explore the involvement of those key immune cells in the progression of AAA, the composition of immune cells between normal and AAA groups, as well as eAAA and rAAA subgroups, was compared receptively. In GSE57691 cohort, the CIBERSORTx results suggested that relative to the control group, naive CD4 T cells (*p* = 0.033), T cells follicular helper (Tfh) (*p* = 0.007), and activated dendritic cells (*p* = 0.004) were more abundant in AAA group, while memory resting CD4 T cells (*p* = 0.015), and M2 macrophages (*p* = 0.041) were more prevalent in control group than in AAA group (Figure 5A). Similar results (activated dendritic cells [*p*  < 0.001], M2 macrophages [*p* = 0.02]) were also observed in the GSE98278 cohort (eAAA subgroup vs. rAAA subgroup) (Figure 6A).

The ssGSEA results demonstrated that activated dendritic cells (*p* = 0.007), immature dendritic cells (*p* = 0.013), Tfh (*p* = 0.013), and IL (*p* = 0.01) were relatively richer in AAA group than control subgroup, while macrophages (*p* = 0.048) were lower in AAA group (Figure 5B). Similar results (activated dendritic cells [*p* = 0.036], macrophages [*p* = 0.014]) were also observed in the GSE98278 cohort (eAAA subgroup vs. rAAA subgroup) (Figure 6B).

Moreover, correlation analysis further demonstrated a significant positive association between PRG and activated dendritic cells (*p*  < 0.001), whereas a considerable negative link was found with M2 macrophages (*p*  < 0.05) in the GSE57691 cohort (control vs. AAA) (Figure 5C). Notably, hub PRGs TNF and CASP1 exhibited significant associations with multiple infiltrating immune cells (Figure 5C). These findings were consistent with those observed in the GSE98278 cohort (eAAA subgroup vs. rAAA subgroup) (Figure 6C).

### 3.6. Discovery of Potential Mechanisms in the Development and Progression of AAA and Its Relationship With Hub PRGs/PRG Classifier

**Figure 7:**
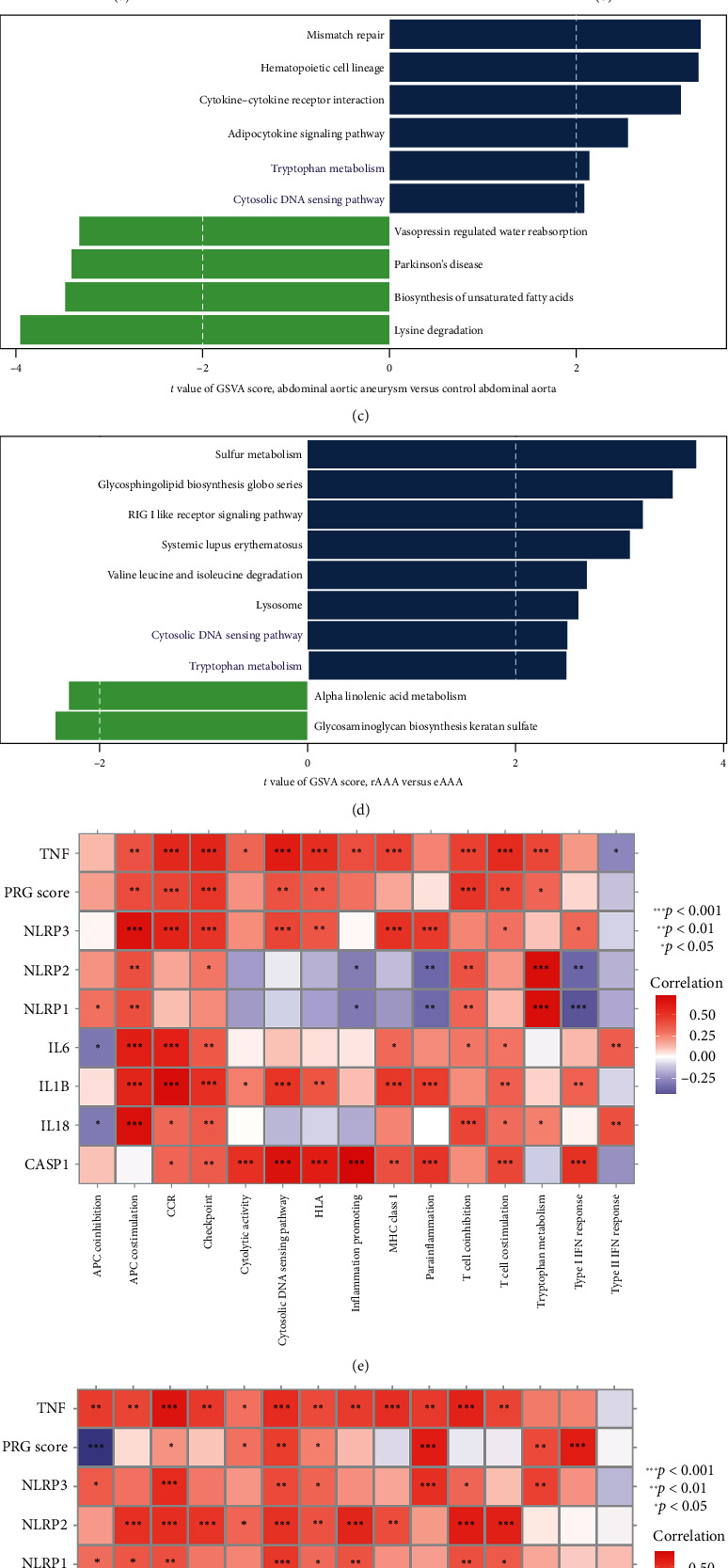
Comprehensive analysis of molecular characteristics during AAA and its relationship with hub PRGs/PRG classifier in GSE57691 and GSE98278 datasets. Comparison of predefined biological processes between normal and AAA groups in GSE57691 dataest (A), as well as eAAA and rAAA subgroups in GSE98278 dataset (B) based on ssGSEA algorithm. *p* value was calculated using the Wilcoxon test. Comparison of KEGG gene sets from Molecular Signatures Database between normal and AAA groups in GSE57691 dataest (C), as well as eAAA and rAAA subgroups in GSE98278 dataset (D) based on gene set variation analysis (GSVA). *t* value and *p* value was calculated using the limma R package. The heatmap plot depicted correlation between hub PRGs/PRG classifier and critical biological pathways in GSE57691 (E) and GSE98278 (F) datasets. Correlation coefficient and *p* value were calculated by Spearman's correlation analysis. The asterisks indicate a statistically significant *p* value calculated using the Spearman's correlation analysis (*⁣*^*∗*^*p*  < 0.05; *⁣*^*∗∗*^*p*  < 0.01; *⁣*^*∗∗∗*^*p*  < 0.001). AAA, abdominal aortic aneurysm; CCR, cytokine–cytokine receptor interaction; eAAA, elective stable AAA; IL, infiltrating lymphocyte; KEGG, Kyoto Encyclopedia of Genes and Genome; PRG, pyroptosis-related gene; rAAA, rupture AAA; ssGSEA, single-sample gene set enrichment analysis; TNF, tumor necrosis factor.

To explore possible biological pathways between normal and AAA groups, as well as eAAA and rAAA subgroups, ssGSEA and GSVA were executed to examine the enrichment score of predefined biological processes and KEGG gene sets, respectively. As for predefined biological processes, it was revealed that antigen-presenting cells (APCs) costimulation, cytokine–cytokine receptor interaction (CCR), human leukocyte antigen (HLA), T cell costimulation, checkpoint, and major histocompatibility complex (MHC) class I exhibited enrichment in the AAA group than in the control group (Figure 7A). Moreover, CCR, HLA, parainflammation, and Type I interferon (IFN) responses exhibited enrichment in the rAAA subgroup relative to the eAAA subgroup (Figure 7B). In terms of KEGG gene sets, the analysis revealed that compared to the normal group, several KEGG gene sets were primarily involved in the AAA group, including mismatch repair, hematopoietic cell lineage, CCR, adipocytokine signaling pathway, cytosolic deoxyribonucleic acid (DNA) sensing pathway, and tryptophan metabolism (Figure 7C). Furthermore, sulfur metabolism, glycosphingolipid biosynthesis globo series, RIG I like receptor signaling pathway, valine, leucine, and isoleucine degradation, as well as tryptophan metabolism and cytosolic DNA sensing pathway were primarily involved in the rAAA subgroup (Figure 7D).

Furthermore, correlation analysis revealed significant positive associations between PRG and CCR, HLA, tryptophan metabolism, and the cytosolic DNA sensing pathway in the GSE57691 cohort (control vs. AAA) (Figure 7E), as well as in the GSE98278 cohorts (eAAA subgroup vs. rAAA subgroup) (Figure 7F). It is noteworthy that eight hub PRGs were considerably linked to these pivotal molecular pathways, suggesting that pyroptosis may interact with them to mediate the occurrence and development of AAA.

### 3.7. Validation Expression of the Hub PRGs by qRT-PCR, WB, and ELISA

**Figure 8:**
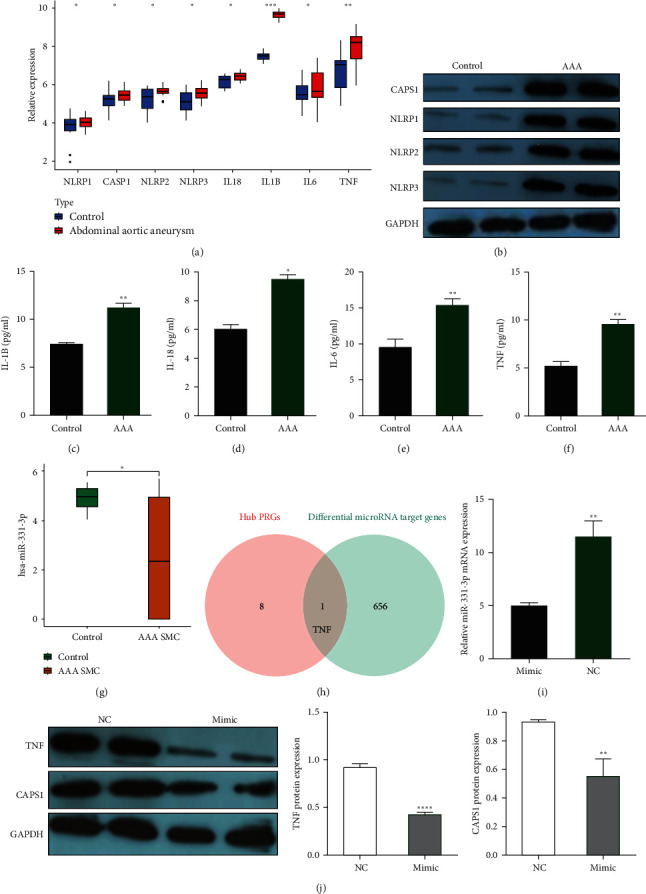
Detection of expression level of the hub PRGs by qRT-PCR, WB, and ELISA as well as construction and validation a miRNA/hub pyroptosis-related molecular regulatory axis. (A) Differential mRNA expression of hub PRGs between AAA patients and control by qRT-PCR. The asterisks indicate a significant statistical *p* value calculated using the Wilcoxon test (*⁣*^*∗*^*p*  < 0.05; *⁣*^*∗∗*^*p*  < 0.01; *⁣*^*∗∗∗*^*p*  < 0.001). (B) The protein expression levels of CASP1, NLRP1, NLRP2, and NLRP3 between AAA patients and control were assessed by WB. Comparison of the protein expression levels of IL1B (C), IL18 (D), IL6 (E), and TNF (F) between AAA patients and control using ELISA detection. The asterisks indicate a significant statistical *p* value calculated using the Wilcoxon test (*⁣*^*∗*^*p*  < 0.05; *⁣*^*∗∗*^*p*  < 0.01; *⁣*^*∗∗∗*^*p*  < 0.001). (G) Comparison of the mRNA expression levels of hsa-miR-331-3p between AAA SMC and control. The asterisks indicate a significant statistical *p* value calculated using the Wilcoxon test (*⁣*^*∗*^*p*  < 0.05; *⁣*^*∗∗*^*p*  < 0.01; *⁣*^*∗∗∗*^*p*  < 0.001). (H) Venn diagrams presents the genes shared by 90 DEmiRNAs target genes and 8 hub PRGs. (I) Measurement of miR-331-3p expression in MOVAS cells transfected with miR-331-3p mimic and NC vectors by RT-qPCR. The asterisks indicate a significant statistical *p* value calculated using the Wilcoxon test (*⁣*^*∗*^*p*  < 0.05; *⁣*^*∗∗*^*p*  < 0.01; *⁣*^*∗∗∗*^*p*  < 0.001). (J) TNF-1 and CASP1 protein level were measured by WB assay in MOVAS cells transfected with miR-331-3p mimic and NC vectors. The asterisks indicate a significant statistical *p* value calculated using the Wilcoxon test (*⁣*^*∗*^*p*  < 0.05; *⁣*^*∗∗*^*p*  < 0.01; *⁣*^*∗∗∗*^*p*  < 0.001). AAA, abdominal aortic aneurysm; DEmiRNAs, differentially expressed microRNAs; ELISA, enzyme-linked immunosorbent assay; IL, infiltrating lymphocyte; NC, negative control; PRG, pyroptosis-related gene; qRT-PCR, quantitative real-time-polymerase chain reaction; TNF, tumor necrosis factor; WB, western blot.

To further confirm the eight hub PRGs expression levels, qRT-PCR, WB, and ELISA were executed in human AAA and normal specimens. The mRNA expression levels of CASP1, IL1B, IL18, IL6, NLRP1, NLRP2, NLRP3, and TNF were significantly upregulated in AAA than in normal specimens (Figure 8A). WB and ELISA also revealed significant increases in the protein expression levels of CASP1, NLRP1, NLRP2, NLRP3 (Figure 8B), IL1B (Figure 8C), IL18 (Figure 8D), IL6 (Figure 8E), and TNF (Figure 8F) in AAA tissues compared to normal tissues.

### 3.8. Construction of the miRNA/Pyroptosis-Related Molecular Regulatory Axis and Validation *In Vitro*

Differential expression analysis revealed a total of 90 DEmiRNAs in the GSE62179 dataset, among which hsa-miR-331-3p exhibited significant downregulation in AAA SMC compared to normal specimens (Figure 8G). Furthermore, predictions from the TargetScan database identified 656 mRNAs as potential targets of the 90 DEmiRNAs. By intersecting the target genes of DEmiRNAs with the eight hub PRGs (Figure 8H), only TNF was identified, establishing its corresponding miRNA as hsa-miR-331-3p. Gain-of-function assays were conducted to validate the reliability of the hsa-miR-331-3p/TNF interaction axis in AAA. Given the reduced expression levels of miR-331-3p in AAA, the miR-331-3p mimic and NC were transfected into MOVAS cells. Results indicated an approximately 2.5-fold increasing in miR-331-3p levels in the mimic groups than the NC groups (Figure 8I). Moreover, the protein expression levels of pyroptosis-related molecules (TNF and CASP1) were assessed via WB. The mimic group exhibited lower protein levels of TNF and CASP1 than the NC group (Figure 8J).

## 4. Discussion

The present research integrated datasets of mRNAs (GSE7084, GSE57691, and GSE98278) and miRNAs (GSE62179) to comprehensively investigate the potential implications of PRGs and their upstream miRNA in the formation and rupture of AAA. Utilizing four machine learning algorithms, the PRG classifier was developed, incorporating eight hub PRGs (CASP1, IL1B, IL18, IL6, NLRP1, NLRP2, NLRP3, and TNF). Validation through qRT-PCR, WB, and ELISA experiments confirmed the mRNA and protein expression of these hub PRGs in human AAA samples. The PRG classifier exhibited excellent diagnostic values, clinical decision, and discrimination performance and displayed substantial positive associations with the risk factors of the AAA (hypertension history, diastolic pressure, triglyceride, and aneurysm diameter) in GEO and clinical cohorts. Moreover, PRG classifier/PRGs showed significant correlations with M2 macrophage infiltration, dendritic cell activation, cytosolic DNA sensing pathway, and tryptophan metabolism, suggesting their potential roles in mediating AAA occurrence and rupture. Importantly, this study elucidated a hsa-miR-331-3p/TNF regulatory axis, demonstrating that overexpression of miR-331-3p led to a considerable downregulation of TNF and CASP1 protein levels. These outcomes offer novel insights into the potential pathological mechanisms underlying AAA formation and rupture, shedding light on the roles of pyroptosis and miRNA in AAA pathogenesis, along with their potential clinical implications.

Pyroptosis, recognized as an inflammatory type of programed cell death, has become a central area of research focus. Its crucial involvement has been noted across various domains, including infectious diseases, tumor immunity, neurological diseases, and cardiovascular diseases [25]. Recently, increasing research has demonstrated that pyroptosis (such as, NLRP3, Caspase-1, GSDMD) mediates the formation of AAA by leading to the loss of VSMC [11, 12]. Nonetheless, the potential roles of pyroptosis molecules, such as NLRP1, NLRP2, TNF, and so on, in AAA are still largely unknown. The present study collected 33 PRGs and comprehensively evaluated their expression pattern, biological functions, and clinical application in the formation and development of AAA. Interestingly, preliminary explorations revealed a predominant upregulation of PRGs in AAA, along with a considerable enrichment of the pyroptosis pathway. Furthermore, the distinct expression patterns of these PRGs allowed for the complete differentiation of AAA patients from control subjects. These findings underscore the pivotal role of dysregulated PRG expression in the onset of AAA. Considering these results, there has been a strong interest in an in-depth understanding of pyroptosis molecules in AAA. Crucially, the specific miRNAs involved in regulating pyroptosis in AAA have yet to be elucidated. To our knowledge, this research represents the first comprehensive investigation into pyroptosis-related molecules across multiple AAA transcriptomes, combined with a clinical cohort analysis. Leveraging a combination of four machine learning approaches, this study aimed to identify and validate novel biomarkers and predictive models with potential clinical significance. It elucidated the crosstalk between pyroptosis and immune cell infiltration, molecular pathways, and upstream miRNA to provide new insight into AAA formation and rupture.

In recent years, machine learning techniques have gained prominence as a novel approach for analyzing large-scale medical data [26], capturing nonlinear relationships, and extracting nuanced information from continuous variables, thereby facilitating the identification of critical factors and improving prediction accuracy. This capability is lacking in traditional predictive models based on Cox or logistic regression analysis in AAA [27]. Recently, Fu et al. [28] applied RF and SVM to construct models based on m6A RNA methylation regulators, respectively. However, their approach lacked the meticulous screening of feature variables, optimization of model coefficients, and external verification of models. This study applied a more rigorous variable selection approach, incorporating LASSO, RF, and SVM in two GEO cohorts to identify the most relevant predictors to construct a model in order to avoid data leakage and overfitting. This research introduced ANN to optimize the weight of variables in AAA, which is a methodology previously used in tumor prediction models to improve predictive power [29]. This strategy enables precise quantification for individual AAA patients, thereby enhancing the predictive performance and generalizability of the model and facilitating tailored treatment strategies.

By integrating multiple machine learning approaches, eight key PRGs were identified, which were used to develop a PRG classifier. The findings from qRT-PCR, WB, and ELISA experiments confirmed that the mRNA and protein expression levels of the hub PRGs (CASP1, IL1B, IL18, IL6, NLRP1, NLRP2, NLRP3, and TNF) were considerably upregulated in human AAA samples compared to normal samples. These results align with several observational studies that have reported elevated mRNA expression levels of NLRP3, Caspase-1, and *IL-1β* in circulating leukocytes of AAA patients in contrast to non-AAA patients [11, 30]. The PRG classifier exhibited outstanding predictive performance with AUC exceeding 0.90 in both GEO datasets. Moreover, it effectively differentiated between large and small AAA, as well as between rAAA and eAAA, along with distinguishing between large and intermediate AAA. DCA results demonstrated that treatment decisions based on the PRG classifier yielded greater net benefits than those based on the approach of treating either all patients or none. This suggests that the PRG classifier holds promise for diagnosing AAA, assessing its severity, and informing clinical decision-making. Commonly recognized risk factors for AAA encompass male gender, age over 65, smoking, hypertension, and dyslipidemia [31]. In the clinical cohorts, individuals in the high PRG subgroup exhibited a trend toward larger aneurysm diameter, higher triglyceride levels, greater height, increased weight, and elevated diastolic pressure, although these differences did not reach statistical significance. Moreover, elevated PRG scores were observed in male and hypertensive patients, while smokers tended to exhibit higher PRG scores, although this trend did not reach statistical significance. These negative results are attributed to the small sample size and need to be explored in a large multicenter cohort. Taken together, the present PRG classifier could serve as an effective tool for the diagnosis and determination of risk stratification in AAA. The superior performance of the model in this study can be attributed to several factors. First, the effective combination of multiple biological markers proved more effective than relying on a single biomarker alone. Second, considering the inherent heterogeneity of AAA, machine learning techniques coupled with transcriptomics allow for a comprehensive understanding and management of disease heterogeneity. Lastly, the selection of biomarkers from a pathophysiological standpoint enhances the predictive power of the model, ensuring its clinical relevance and applicability.

The pathophysiological processes of AAA encompass inflammatory cell infiltration, degradation of elastic and collagen fibers, heightened oxidative stress, arterial wall defects, and death of smooth muscle cells (SMCs) [32]. In the initial phases of the disease, immune cells like lymphocytes, macrophages, neutrophils, and natural killer (NK) cells infiltrate and accumulate in the blood vessels and surrounding tissues. This leads to a cascade of inflammatory responses within the vascular wall, diminishing its stability and ultimately triggering VSMC death [32]. In this study, during AAA formation and rupture, a considerable elevation in dendritic cell activation and a notable decrease in M2 macrophage infiltration were observed. As AAA progresses, M1 macrophages instigate chronic inflammation, hindering the repair of the injured aorta. Conversely, M2 macrophage polarization diminishes inflammation, facilitating wound healing and leading to the downregulation of M1 macrophages [33]. In the Ang II-induced AAA mouse model, the chemokine C–C motif ligand has been observed to contribute to AAA development and pathogenesis by facilitating the polarization of macrophages from M2 to M1 [34]. These findings align with our study, which suggests that the reduced infiltration of M2 macrophages might play a role in the formation and rupture of AAA. However, the impact of dendritic cell activation on AAA remains relatively understudied compared to other immune cell types. Yan et al. [35] demonstrated that in the aneurysmal process, DNA–cathelicidin-related antimicrobial peptide (CRAMP) complexes released along with neutrophil extracellular traps (NETs) could potentially be internalized by dendritic cells. This uptake may trigger dendritic cell activation, leading to the production of type I IFN and instigating inflammatory responses. Notably, their study showed that depletion of dendritic cells abolished T cell recruitment and activation, matrix metalloproteinases activity, and ultimately hindered aneurysm development. These findings strongly suggest that NET-dependent priming of dendritic cell activation is essential for AAA formation in their model. Notably, this study revealed a notable correlation between the PRG classifier/hub PRGs and M2 macrophage infiltration, as well as dendritic cell activation. This suggests that the interplay between pyroptosis and these immune processes might be involved in the onset and rupture of AAA. Currently, the precise mechanisms underlying how pyroptosis interacts with macrophage polarization and dendritic cell activation to lead to AAA formation and rupture remain elusive. Further investigation in this direction is warranted to unveil new therapeutic avenues for treating AAA.

ssGSEA and GSVA functional enrichment analyses were performed to elucidate the underlying biological mechanisms of AAA. The ssGSEA results revealed that immune and inflammation-related pathways (CCR, HLA, T cell costimulation, parainflammation, and Type I IFN response) were predominantly enriched in AAA formation and rupture, consistent with previous studies [36]. Furthermore, GSVA results utilizing KEGG gene sets revealed significant involvement of tryptophan metabolism and the cytosolic DNA sensing pathway in the formation and rupture of AAA. Tryptophan, as an essential amino acid, is critically involved in protein synthesis, thereby contributing to normal cellular homeostasis. Growing evidence suggests that dysregulated tryptophan metabolism contributes to aortic inflammation and aortic disease phenotypes [37]. In studies utilizing both apolipoprotein e (Apoe^–/–^) and indoleamine 2,3-dioxygenase (IDO) (/IDO^–/–^) AAA mice models, researchers established a causative relationship between aberrant tryptophan metabolism and AAA. Specifically, 3-HAA, a metabolite of tryptophan, activated the nuclear factor-kB transcription factor, resulting in the upregulation of matrix metallopeptidase 2 expression in VSMCs, thereby promoting the formation of AAA [38]. Cytosolic DNA sensors constitute a group of pattern recognition receptors (PRRs) with a shared function of detecting intracellular microbial DNA, thus initiating the innate immune response. These sensors have been shown to be pivotal in various biological processes, including microbial infection, chronic inflammation, cancer progression, and organ degeneration. They represent a promising area of investigation for elucidating the underlying mechanisms of multiple disease pathogenesis [39]. Nonetheless, the involvement of the cytosolic DNA sensing pathway in AAA formation and rupture is nascent. A recent investigation revealed a substantial increase in DNA damage and the activation of the cytosolic DNA sensing adaptor STING (stimulator of IFN genes) signaling pathway in aortic tissues obtained from patients diagnosed with sporadic thoracic aortic aneurysm and dissection [40]. Crucially, this study uncovered a significant association between the PRG classifier/hub PRGs and the tryptophan metabolism and cytosolic DNA sensing pathway. This suggests that pyroptosis and their interplay could potentially drive the onset and rupture of AAA. At present, the specific mechanism underlying AAA formation and rupture mediated by the interaction between pyroptosis and pathways such as tryptophan metabolism and the cytosolic DNA sensing pathway remains unclear. Further research in this direction is imperative to unveil novel strategies for the treatment of AAA.

One significant discovery of this study was the identification of the miR-331-3p/TNF regulatory axis, which holds promise as a novel therapeutic target for AAA. TNF, known for its inflammatory properties, is recognized for its pivotal role in linking various types of programed cell death due to its intricate signaling pathways [41]. TNF exerts complex cell death signaling by interacting with TNF receptor 1 [42], leading to diverse outcomes such as apoptosis, pyroptosis, and necroptosis, depending on the assembly of TNF complexes (TNF Complex IIa, IIb, and IIc). Previous research has shown that inhibiting TNF-*α* can impede AAA progression by diminishing the expression of pro-inflammatory cytokines and adhesion molecules, thereby restricting macrophage infiltration [43]. In our study, TNF, identified as a pyroptosis-related molecule, exhibited significantly increased mRNA expression and protein levels across two GEO datasets and clinical cohorts. Elevated TNF expression showed a significant positive correlation with dendritic cell activation and the cytosolic DNA sensing pathway. The regulatory role of its upstream miRNA in the regulation of AAA pyroptosis is yet to be elucidated. Based on 90 DEmiRNAs and eight hub PRGs in AAA through the TargetScan dataset, a hsa-miR-331-3p/TNF regulatory axis was constructed. miR-331-3p, a member of the miR-331 family, is situated on chromosome 12q22 and has predominantly been studied in the context of cancer research. miR-331-3p has been implicated in regulating cellular progression in multiple cancer types, encompassing hepatocellular carcinoma, prostate cancer, and non-small cell lung cancer (NSCLC) [44–46]. The increased expression of miR-331-3p has been shown to eliminate the tumor-suppressing function of circRASSF5 and sponge 3′UTR of PHLPP and enhance the protein level of PHLPP, ultimately leading to the promotion of HCC cell growth [44]. miR-331-3p has been proven to be a promoting tumor regulator in prostate cancer, which acts as a mediator of ErbB-2 expression and PI3K/AKT signaling [45]. Moreover, miR-331-3p overexpression has been shown to counteract the inhibitory effect of AC005332.7 on ferroptosis in OGD-induced AC16 cells, exacerbating cardiomyocyte injury in acute myocardial infarction [47]. In this study, a notable upregulation of hsa-miR-331-3p expression was observed in AAA SMCs relative to normal specimens. However, the regulatory impact of miR-331-3p on AAA pyroptosis remained unclear. Through loss-of-function assays conducted in vitro using MOVAS cells, it was observed that increasing miR-331-3p expression led to a substantial reduction in the protein levels of TNF and CASP1. These outcomes highlight that miR-331-3p might alleviate AAA pyroptosis and could potentially serve as a novel therapeutic target for AAA treatment.

In comparison to earlier studies, this research introduces several distinctive features. First, a comprehensive assessment and validation of the expression patterns, biological functions, and clinical relevance of 33 PRGs in both GEO datasets and clinical cohorts were conducted, shedding light on their involvement in AAA formation and progression. Second, a novel approach was employed by integrating four machine learning techniques (LASSO, RF, SVM, and ANN) in two independent AAA GEO cohorts. This facilitated the identification of robust hub genes and optimization of model coefficients, ultimately enhancing the predictive performance of the models. Third, the study uncovered the intricate crosstalk between pyroptosis and key biological pathways such as macrophage polarization, dendritic cell activation, tryptophan metabolism, and the cytosolic DNA sensing pathway, offering new insights into the mechanisms underlying AAA pathogenesis. Lastly, the regulatory axis of hsa-miR-331-3p/TNF was elucidated and validated, potentially opening up new avenues for AAA treatment and deepening our comprehension of its pathogenesis. Despite the considerable outcomes, this research also has limitations. First, the small sample size in both the GEO and clinical cohorts suggests that the generalizability of the model needs to be verified in larger prospective cohorts. Second, while various algorithms and datasets were employed to validate the findings, methods like CIBERSORTx deconvolution, ssGSEA, and GSVA with metagenes may not accurately assess immune cell subpopulations and biological pathways. Future studies could benefit from single-cell RNA-Seq methods or fluorescence-activated cell sorting for more precise evaluations. Lastly, additional in vitro and in vivo investigations are warranted to elucidate the molecular mechanism and confirm the role of miR-331-3p in regulating AAA pyroptosis.

## 5. Conclusion

Utilizing eight PRGs identified through four machine learning algorithms, a PRG classifier was developed and clinically validated for potential diagnosing and assessing the severity of AAA. Furthermore, the identification of the cytosolic DNA sensing pathway and the hsa-miR-331-3p/TNF regulation axis could represent crucial targets for AAA treatment, offering insights into the underlying pathogenesis of the disease.

## Figures and Tables

**Table 1:** Demographic and clinical characteristics of abdominal aortic aneurysm patients.

Variables	Patients (*n* = 15)
Male sex, no. (%)	11 (73.3)
Age, median (IQR), years	59 (37–65)
Height, mean (SD), (m)	1.71 (0.12)
Weight, median (IQR), (kg)	66 (60–72)
BMI, mean (SD), (kg/m^2^)	1.71 (0.12)
Hypertension, no. (%)	7 (46.7)
Smoking, no. (%)	4 (26.7)
Fasting blood glucose, median (IQR), (mmol/l)	5.23 (4.79–6.17)
Systolic pressure, median (IQR), (mmHg)	125 (120–131)
Diastolic pressure, median (IQR), (mmHg)	80 (70–91)
Triglyceride, median (IQR), (mmol/l)	1.24 (1.10–1.61)
Cholesterol, mean (SD), (mmol/l)	4.65 (0.81)
Aneurysm diameter, median (IQR), (mm)	61 (50–69)

Abbreviations: BMI, body mass index; IQR, interquartile range; SD, standard deviation.

## Data Availability

The original data presented in the study are included in the article/supporting information. Further inquiries can be directed to the corresponding author. The codes underlying this article are available in GitHub at https://github.com/GenglongLiu/AAA-codes.

## Nomenclature

AAA: Abdominal aortic aneurysm
miRNA: micorRNA
PRGs: Pyroptosis-related genes
qRT-PCR: Quantitative real-time-polymerase chain reaction
WB: Western blot
ELISA: Enzyme-linked immunosorbent assay
PCA: Principal component analysis
GEO: Gene expression omnibus
ROC: Receiver operating characteristic
AUC: Area under curve
eAAA: Elective stable AAA
rAAA: Rupture AAA
VSMCs: Vascular smooth muscle cells
ncRNAs: Noncoding RNAs
ssGSEA: Single-sample gene set enrichment analysis
DEGs: Differentially expressed genes
LASSO: Least absolute shrinkage and selection operator
RSF: Random survival forest
SVM: Support vector machine
ANN: Artificial neural network
DCA: Decision curve analysis
GSVA: Gene set variation analysis
KEGGs: Kyoto Encyclopedia of Genes and Genomes
BMI: Body mass index
FBG: Fasting blood glucose
DEME: Dulbecco's modified Eagle's medium
NC: Negative control
CRAMP: Cathelicidin-related antimicrobial peptide
NETs: Neutrophil extracellular traps
Apoe: Apolipoprotein e
IDO: Indoleamine 2,3-dioxygenase
HCC: Hepatocellular carcinoma.

## Supporting Information

Supporting Information 1 Thirty-three pyroptosis-related genes (PRGs) were compiled from published studies.

Supporting Information 2 Gene-specific polymerase chain reaction (PCR) primers are provided in the present study.

Supporting Information 4 Figure S1. Correlation of PRG classifier with clinical characteristics in hospital cohort.

Supporting Information 5 Figure S2. Correlation of PRG classifier with clinical characteristics in hospital cohort.
