# Supplementary figures and images for: Construction of the miRNA/Pyroptosis-Related Molecular Regulatory Axis in Abdominal Aortic Aneurysm: Evidence From Transcriptome Data Combined With Multiple Machine Learning Approaches Followed by Experiment Validation
Source: J Immunol Res. 2024 Oct 30;2024:1429510. doi: 10.1155/2024/1429510 (PMC11540895; doi:10.1155/2024/1429510)

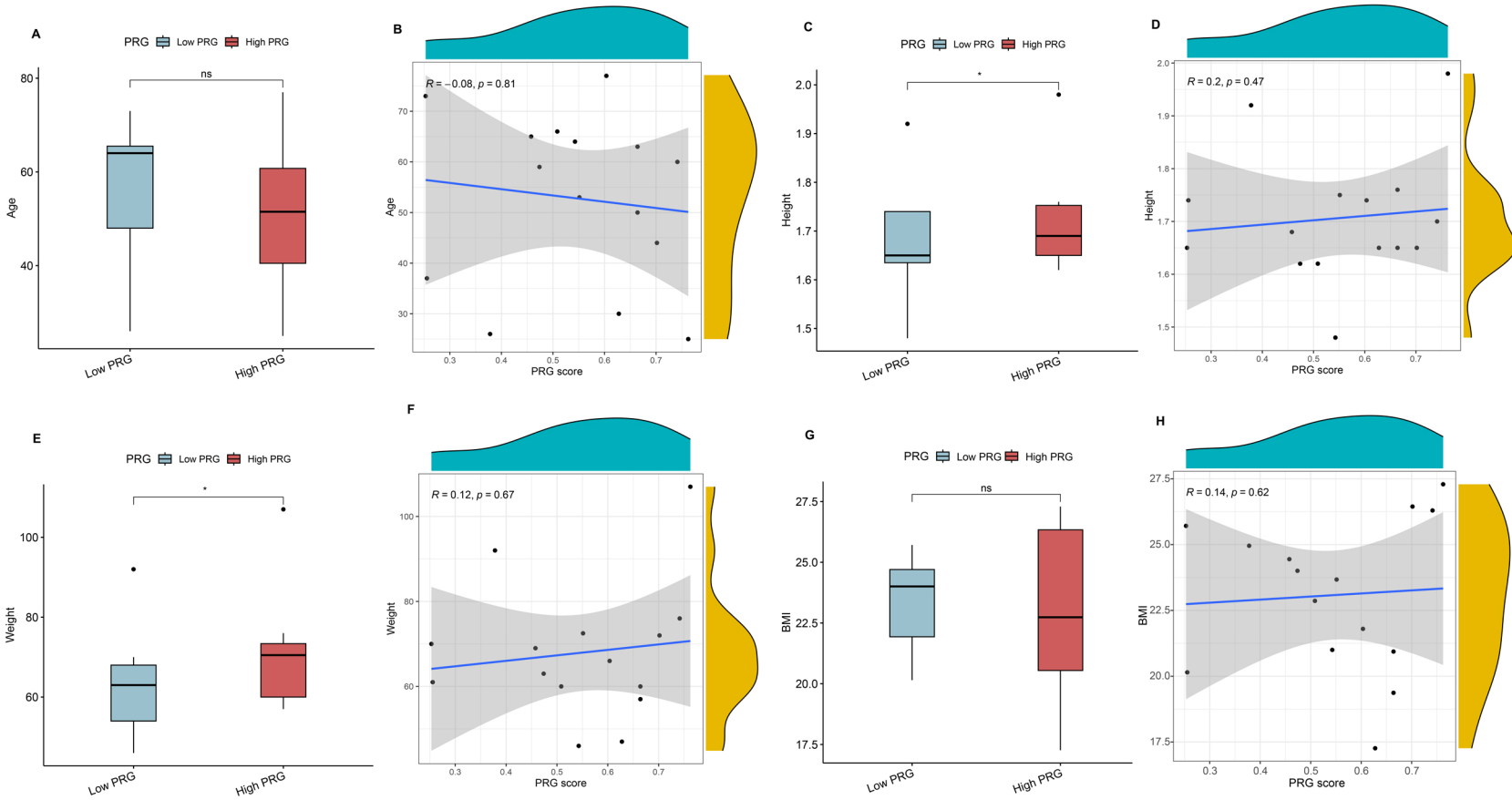

Supplement: Supporting Information 4 — Figure S1. Correlation of PRG classifier with clinical characteristics in hospital cohort. [file 1429510.f4.pdf]

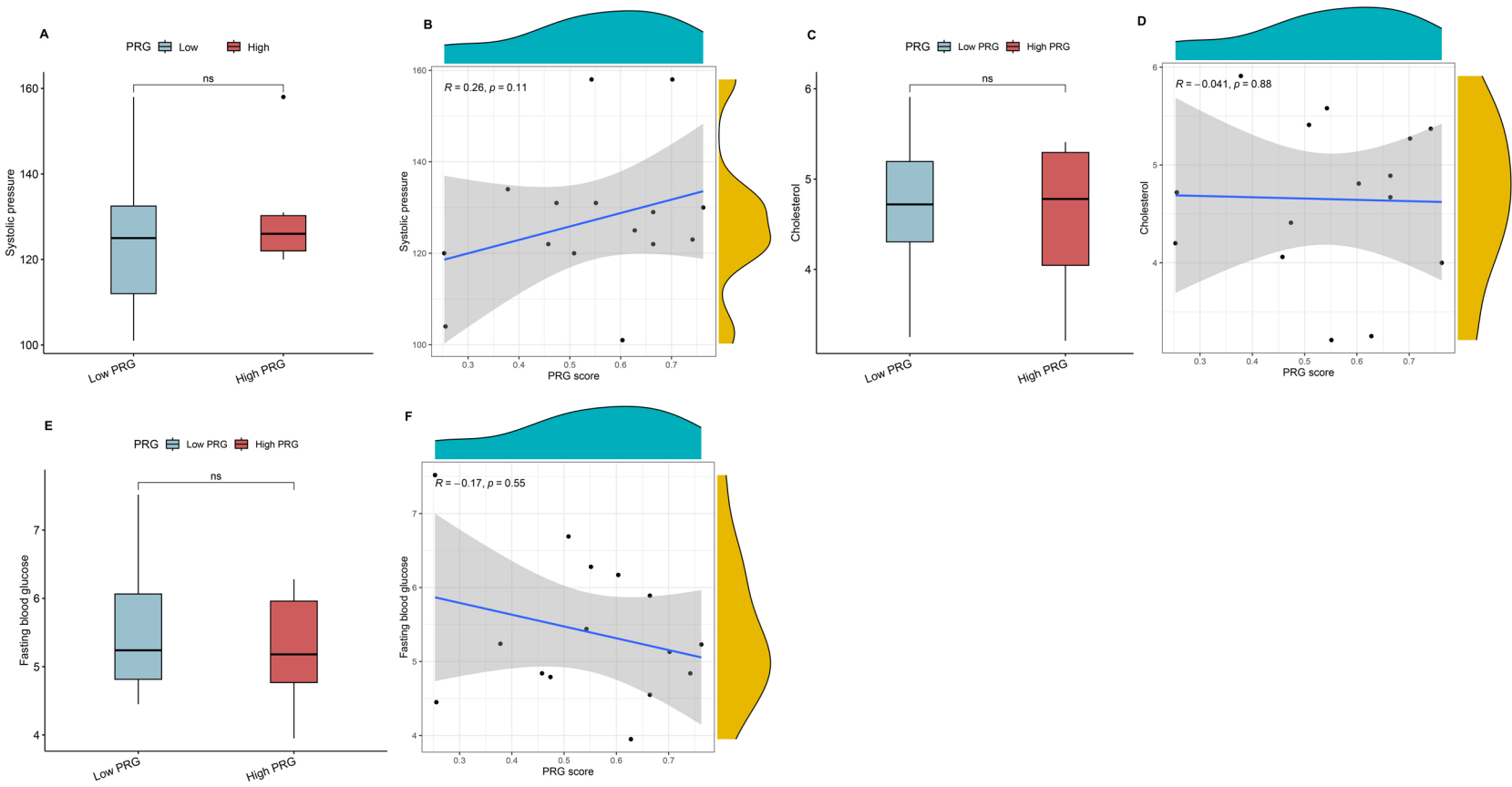

Supplement: Supporting Information 5 — Figure S2. Correlation of PRG classifier with clinical characteristics in hospital cohort. [file 1429510.f5.pdf]
